# Dominance Norms and Data for Spoken Ambiguous Words in British English

**DOI:** 10.5334/joc.194

**Published:** 2022-01-06

**Authors:** Rebecca A. Gilbert, Jennifer M. Rodd

**Affiliations:** 1 University College London, London, UK; 2 MRC Cognition and Brain Sciences Unit, University of Cambridge, Cambridge, UK; 3 Department of Experimental Psychology, University College London, London, UK

**Keywords:** Auditory word processing, Semantics, Speech perception

## Abstract

Words with multiple meanings (e.g. bark of the tree/dog) have provided important insights into several key topics within psycholinguistics. Experiments that use ambiguous words require stimuli to be carefully controlled for the relative frequency (dominance) of their different meanings, as this property has pervasive effects on numerous tasks. Dominance scores are often calculated from word association responses: by measuring the proportion of participants who respond to the word ‘bark’ with dog-related (e.g. “woof”) or tree-related (e.g. “branch”) responses, researchers can estimate people’s relative preferences for these meanings. We collated data from a number of recent experiments and pre-tests to construct a dataset of 29,542 valid responses for 243 spoken ambiguous words from participants from the United Kingdom. We provide summary dominance data for the 182 ambiguous words that have a minimum of 100 responses, and a tool for automatically coding new word association responses based on responses in our coded set, which allows additional data to be more easily scored and added to this database. All files can be found at: *https://osf.io/uy47w/*.

## Introduction

Most common wordforms correspond to more than one meaning: at least 80% of common English words have more than one dictionary entry (Rodd, Gaskell, & Marslen-Wilson, 2002). The way in which listeners and readers access the intended meanings of ambiguous words within sentence contexts is therefore a key question within psycholinguistics (Duffy, Kambe, & Rayner, 2001; Twilley & Dixon, 2000). In addition, studies using ambiguous words have provided important insights into several issues of broad theoretical importance, including the structure and plasticity of the adult mental lexicon (Rodd, 2020).

It has long been known that the relative frequency (dominance) of the meanings of an ambiguous word has a substantial, pervasive influence on both the processing and learning of these meanings, with higher-frequency meanings being more readily available and associated with less processing load than lower-frequency meanings (Vitello & Rodd, 2015). Researchers who wish to use ambiguous words in their experiments must therefore routinely control for meaning dominance. This need to quantify meaning dominance is not straightforward as even within a particular linguistic community an individual’s meaning preferences will vary according to their specific, idiosyncratic linguistic experiences. Numerous studies using word-meaning priming paradigms have shown that participants’ meaning preferences can be influenced by having encountered a particular ambiguous word within the previous minutes/hours (Betts, Gilbert, Cai, Okedara, & Rodd, 2018; Gaskell, Cairney, & Rodd, 2019; Gilbert, Davis, Gaskell, & Rodd, 2018, 2021; Rodd et al., 2016; Rodd, Lopez Cutrin, Kirsch, Millar, & Davis, 2013). In addition, several studies have found longer term effects of people’s hobbies on their meaning preferences (Eligio & Kaschak, 2020; Rodd et al., 2016; Wiley, George, & Rayner, 2018). However, despite this individual variability, researchers often must rely on published dominance norms to obtain estimates of the dominance of their stimuli that they assume will match, to a greater of lesser extent, the average meaning preferences of the individuals who will participate in their experiments.

At present, there are three major sources of English dominance norms: Twilley et al. (1994), Armstrong, Tokowicz, and Plaut (2012), and Maciejewski and Klepousniotou (2016). The norms provided by Twilley and colleagues are widely used, perhaps because they appear to be the most recent set that uses the word association method with a large set of items (566 homographs). This dataset comes from Canadian participants and relied on the word association method to estimate dominance: they measured, for example, the percentage of people who responded to the word “bark” with dog-related vs. tree-related words (e.g., “dog”, “woof” vs. “tree”, “rough”) and assumed that the relatively frequencies of these responses closely corresponds to the relative availability (i.e. dominance) of the meanings in that group of participants. More recently, Armstrong et al. (2012) introduced a new, and arguably more efficient, method for estimating meaning dominance, called ‘eDom’, in which participants make explicit estimates about the relative frequencies of all of a word’s meanings. They report norms from 544 homonyms based on data collected from US-based participants. Finally, Maciejewski and Klepousniotou provide recent norms for 100 homonyms, which were also collected with the eDom method but from British participants.

Here we add to these useful resources (i) a word association database, containing the actual responses that UK-based participants gave to 243 *spoken* target words, (ii) summary dominance statistics for the 182 ambiguous words that have 100+ responses, and (iii) an R script that can be used to automate the scoring of new responses to the target words included in the database. Word association and eDom methods each have their own strengths and weaknesses for estimating meaning dominance (see Armstrong et al., 2012 for extensive discussion of the relative merits of each). It is currently uncertain which method is optimal, or whether an optimal approach might be to combine the dominance estimates obtained from these complementary methods. The current report uses word association for purely pragmatic reasons: the majority of the data reported here come a large set of previously-published experiments that used word association responses. The aim of these earlier experiments was not to estimate meaning dominance, but instead to answer research questions about the effect of recent linguistic experience on preferred interpretations of ambiguous words. In these word-meaning priming experiments (Betts et al., 2018; Gilbert et al., 2018, 2021; Rodd et al., 2016, 2013), participants are first exposed to ambiguous words in resolved sentence contexts, and are later presented with a word association test as a measure of their preferred interpretation of the ambiguous words. Word association is typically used as the test because it provides a measure of the participants’ interpretation in the absence of any biasing, pre-determined context. While many of the words used in the association test cannot be used for norms because they are primed from the sentence exposures, participants in these studies are also presented with a subset of ambiguous words in an *unprimed* condition, in order to establish a baseline proportion of word association responses related to the primed (sentence-consistent) meaning. Taken together, these ‘unprimed’ word association responses represent a wealth of data about word meaning dominance. The current database makes use of this previously untapped resource by collating these data and standardising the way in which the responses were coded. To maximise the number of ambiguous words in the database for which there was sufficient data to adequately estimate meaning dominance, we collected additional word association data from 100 participants for 149 of the ambiguous words.

This new database differs from the previous dominance databases in several key ways. First, the aforementioned existing norms have all used *visual* presentation of the ambiguous words. These norms are likely to be misleading when selecting stimuli for studies of spoken language: many words acquire additional ambiguity when presented in the auditory modality. For example, the word “sew” in its printed form usually refers either to “making stitches with a needle and thread” or “planting seeds in the earth”, but in spoken form can also refer to the very common adverb “so”. In addition, researchers studying spoken language often choose to increase the number of stimuli in their experiments by including non-homographic homophones such as “night/knight” and “profit/prophet” in their experiments, which are only (or primarily) ambiguous in spoken form and therefore unlikely to be included in norms that use visual presentation. Also, while our own work on word-meaning learning suggests that comprehenders do not always track word usage in a modality-specific way (at least not following a single recent exposure; Gilbert et al., 2018), there may be subtle differences in the ways that words tend to be used in spoken versus written language (see e.g. Berman & Nir, 2010; Hayes, 1988 for evidence of modality effects on a number of measures). Over time and exposures, any systematic differences in word usage could lead to modality effects on word-meaning interpretation. The current database therefore provides useful meaning dominance information for researchers who are interested specifically in spoken language.

Second, the large scale norms provided by Twilley et al. and Armstrong et al. were collected from North American participants, which severely limits their use in experiments with UK-based participants. There are important differences in word usage between North American and British English dialects that will affect the meaning categories and dominances of some words. Not only is it possible for there to be subtle differences in word-meaning frequencies between dialects, but there may also be words that are associated with meanings in one dialect that are not commonly used by another. For example, in British English, “tap” is used to refer to a device that controls the flow of water (commonly called a “faucet” in American English), “spade” can refer to a tool for digging (i.e. “shovel”), and “tip” can refer to a place for trash (i.e. “dump”; see Cai et al., 2017 for more examples). For experiments with British English speakers, it is critical that researchers use meaning dominance estimates that most accurately capture the pattern of word usage for these participants.

Third, word meaning associations and frequencies change over time (Armstrong, Zugarramurdi, Cabana, Valle Lisboa, & Plaut, 2016; Swinney, 1979; Twilley et al., 1994), so the utility of the Twilley et al. norms is especially reduced because they are now over 25 years old. For instance, the ambiguous word ‘post’ has taken on an increasingly common additional social media meaning, while the ‘VHS’ and ‘cassette’ meanings of the word ‘tape’ will have declined in use. In collecting an up-to-date set of norms using the word association method, we can ensure that the most relevant data is used to estimate meaning frequency in contemporary experiments.

A fourth key innovation in the current dataset relates to the construction of meaning categories when coding the responses. One of the challenges in coding word association responses is in deciding whether two different responses should be mapped onto a single meaning category or separated into distinct categories. For some homonymous words (e.g. ‘bark’), this distinction is fairly straightforward because the two meanings are clearly unrelated, resulting in two clear categories of responses. However most words are polysemous in that they have multiple related meanings or ‘senses’ (possibly in addition to unrelated meanings), and any attempt to build a dominance database must make a series of tricky decisions about whether such related senses should be combined into a single meaning category or kept distinct. For example, the word “card” has several highly related senses including “credit card”, “playing card” and “greeting card”. These decisions can have dramatic impacts on dominance estimates. Depending on the specific experimental aims, researchers may wish to know about the relative frequencies of these individual senses, or may prefer them to be combined together into a single, broader meaning category. In the current project we took a pragmatic approach to setting up meaning categories. We used a maximally fine-grained meaning coding system – responses were categorised separately whenever the intended meaning/sense could be reliably distinguished from the others. For example, we determined that the different senses of “card” could easily be identified on the basis of participants’ responses. This coding system will allow researchers to combine and/or exclude these meaning categories to easily re-calculate dominance in a way that is best suited to the aims of their own work.

Finally, in addition to making both the raw and summary data publically available, we have created an automated coding script to assist with the coding of future word association responses. For any given ambiguous word there is typically a set of common responses, with some items producing a very high number of these repeated associates across respondents (e.g. in our data, the words “tree” and “dog” make up about 88% of responses to the word “bark”). For this reason, a large portion of new (uncoded) word association responses can be coded automatically by searching the existing data for the same item-response pair in the coded data set and assigning the same meaning category. This means that manual coding is only required for entirely novel associate responses. This script will improve the efficiency of coding new word association responses and reduce manual coding errors and inconsistencies. All files can be found at: *https://osf.io/uy47w/*.

## Method

### Inclusion of Existing Datasets

The database was initially built from existing datasets. These data came from nine previously published word-meaning priming experiments (Betts et al., 2018; Gilbert et al., 2018, 2021; Rodd et al., 2016, 2013; see ***Table 1***). We also included the data from one unpublished priming study (Betts, 2013) that used a very similar method to Betts et al. (2018, Experiment 1). We selected these datasets because they all contained unprimed word association responses to a set of spoken ambiguous words from similar UK-based populations, and in all cases we had access to the raw data. For all these experiments, we only included responses from trials in which the participant had not previously encountered the ambiguous word during the experiment (i.e. the unprimed conditions). Finally, we included the responses from a stimuli pre-testing study (Warren et al., unpublished) in which the task instructions and participant population was similar to those used in the published papers.

**Table 1:** Sources of the word association data.


NUMBER	EXPERIMENT	EXPERIMENT TYPE	NUMBER OF AMBIGUOUS WORDS	NUMBER OF PARTICIPANTS

1	Betts et al. (2018), Expt. 1	Priming	60	30

2	Betts et al. (2018), Expt. 2	Priming	88	55

3	Betts et al. (2018), Expt. 3	Priming	88	58

4	Betts (2013)	Priming	56	20

5	Gilbert et al. (2018), Expt. 1	Priming	75	78

6	Gilbert et al. (2021), Expt. 1	Priming	55	30

7	Gilbert et al. (2021), Expt. 3	Priming	65	109

8	Rodd et al. (2016), Expt. 2	Priming	88	40

9	Rodd et al. (2013), Expt. 1	Priming	113	29

10	Rodd et al. (2013), Expt. 3	Priming	54	42

11	Warren et al. (unpublished)	Stimuli pre-test	192	25


In all these experiments, the participants were native speakers of British English, aged 18–60. The sound files used differed across the different experiments, but in all cases were spoken by a female speaker of British English. The sound files used for each experiment can be found at *https://osf.io/uy47w/*.

Across all experiments, the instructions in the word association task were to respond with the first related word that came to mind. Participants were given examples of item-response pairs before starting the task. The word association procedure varied slightly across the experiments: in Rodd et al. (2013), participants were asked to first type the spoken ambiguous word into a response box to ensure that it was heard correctly, and in Rodd et al. (2016), participants responded verbally and their responses were later transcribed. Aside from these exceptions, participants responded to the spoken word by typing an associate into a response box on a computer screen.

### Coding Procedure

As with previous dominance norms based on word association data (Twilley et al., 1994) it was necessary to code each response as to which of the word’s meanings it was most likely related. Although all the word association data had already been coded in this way prior to the creation of this database, all items were recoded to ensure that these response classifications consistently mapped onto the specific meaning/sense categories used in the current database.

The first stage was to create an initial list of potential meanings for each ambiguous word based on the classifications used in the previous analyses of these experiments. For example, the word “bark” was initially assigned two possible meanings: “dog noise” and “outer covering of tree”. Each individual response was then coded based on these definitions. In cases where a response was clearly related to a different meaning/sense, then a new definition/code was created. For example, a single participant responded to “bark” with the name “Mozart” suggesting that they had interpreted the target word as “Bach” (a homophone of “bark” for many of our participants) so a third “German Composer” meaning was created.

In many cases, difficult judgements were needed as to whether responses should best be coded as falling within an existing meaning category, or whether an additional (semantically-related) category should be created. For example, the ambiguous word “card” has the related senses of playing cards, greeting cards, and debit/credit cards. As previously discussed, we separated into distinct categories whenever we felt it was possible to do so in a reliable and consistent manner because participants’ responses clearly related to only one of these senses (e.g. “joker”, “birthday”, “swipe”). This approach created a database that is as fine-grained as we felt it was possible to create.

All responses were coded as to which of these meanings they were most closely related to. We assigned a code of 0 to any responses that were ambiguous in their relationship to the different meanings (e.g. “write” in response to the ambiguous word “letter”, which could refer to either the “alphabetic character” or “written correspondence” meaning) or that were not clearly related to any meaning of the word. Responses with a 0 code were considered invalid/uninterpretable and were not included in the estimates of meaning dominance. However, we retained these responses in the data set in order to maintain transparency and consistency, and to aid with the automatic coding of future responses (see Automated Coding Script section).

Responses were coded by a single researcher (RG). In the event that the initial coder was unsure which code to assign, another researcher (JR) reviewed the responses and the two reached consensus. If no consensus could be reached then the response was considered ambiguous and coded as 0. Once there were a large number of coded responses per word, we used a combination of automated and manual coding for the remaining responses.

In order to estimate inter-rater reliability, after all data had been coded, a third researcher coded a random subset of 10% (3,124) of the total responses using our definitions. The third coder was a research assistant with no prior involvement in this study and was given the raw data (item-response pairs) along with our list of meanings/codes, but was blind to the response codes that we assigned. In order to maximize the information obtained from this re-coding, the data were randomly sampled from a subset of the unique item-response pairs. The inter-rater reliability from this sample was 89%. Of the third rater’s response codes that did not match our own, most of these discrepancies (91%) occurred because one rater assigned a code for a specific meaning and the other coded the responses as ambiguous/uninterpretable. The remaining 9% of inter-rater coding discrepancies occurred due to assigning two different valid meaning codes.

### Automated Coding Script

The aim of this script is to automate the coding of new word association responses that are already included in the database and thus do not need to be manually coded. Because some target words tend to elicit only a small number of different responses, this can greatly reduce the workload associated with response coding and ensures the consistency and accuracy of coding for repeated target-response pairs.

Word association data can be automatically coded using the custom R function “scoreWAdata.R” (*https://osf.io/tb3vh/*), which we demonstrate with the example script “automatic_WA_coding_example.R” (*https://osf.io/q8gbj/*) and uncoded example data (*https://osf.io/r46x2/*). The “scoreWAdata” function takes the following input arguments: (1) the new data to be coded, (2) the column name in the new data containing the target word (e.g. “item”), and (3) the column name in the new data containing the participants’ responses to the target word (e.g. “response”). By default, the function will use the raw data file that we provide to score the responses, but this can be changed to any data frame that contains columns for items, responses, and meaning codes. The function checks for identical pairs of target words and responses in the coded data set. Participants’ responses in both the coded and uncoded data are first changed to lowercase before the comparison^1^, and any spaces before or after the responses are ignored. If a match is found, then the response in the new data set is assigned the same code as that in the coded data set, and a flag variable is set to 1 (i.e. ‘automatically coded’). If no match is found, then no code is assigned to the response and the flag variable is set to 0 (i.e. ‘not coded’). Any responses that are flagged with 0 can then be reviewed by the experimenter for manual coding. If there are any target words in the new data set that are not found in the coded data set, then all responses to that cue word are assigned a code of 99 (i.e. ‘item not present in the coded data’). Because the script checks for an exact match, we recommend that the experimenter reviews the responses for any obvious spelling errors and typos in participants’ responses before running this coding script.

After recoding the data from 9 experiments, we tested this script on the existing data using items with at least 100 responses (N = 50). For each item, 80 ‘training’ responses and 20 ‘test’ responses were selected at random. We then ran the script on the 20 test responses using the 80 training responses, and calculated the proportion of test responses that could be successfully coded. This procedure was repeated 100 times for each item, in order to account for the random selection of training and test responses. The proportions of successfully coded responses were then averaged over the 100 repetitions for each item. The mean percentage of successfully coded test responses was 80% across all items, indicating that for any target word with at least 80 existing responses, the script can reduce the workload associated with coding new responses by 80% on average. The usefulness of the script varies across items, with only 62% of responses being successfully coded for the target word ‘craft’ (which reflects a relatively high degree of response variability), whereas 95% of responses were successfully coded for the target word ‘pupil’ (reflecting a relatively low degree of response variability).

### Additional Data Collection

Due to the differences in the target words used across the 11 experiments/pre-tests (see ***Table 1***), there was a wide range of numbers of responses per item: some items were included in virtually all experiments, while others were only included in a single experiment. We note that many of the ambiguous words with relatively few responses (<40) were excluded from subsequent experiments because they had proven problematic, for example because they generated high numbers of ambiguous responses. We nonetheless included the items with lower response counts in the database as this information might still be helpful for researchers, for instance, to supplement their own data collection or reveal any potential issues with response ambiguity.

**Figure 1:**
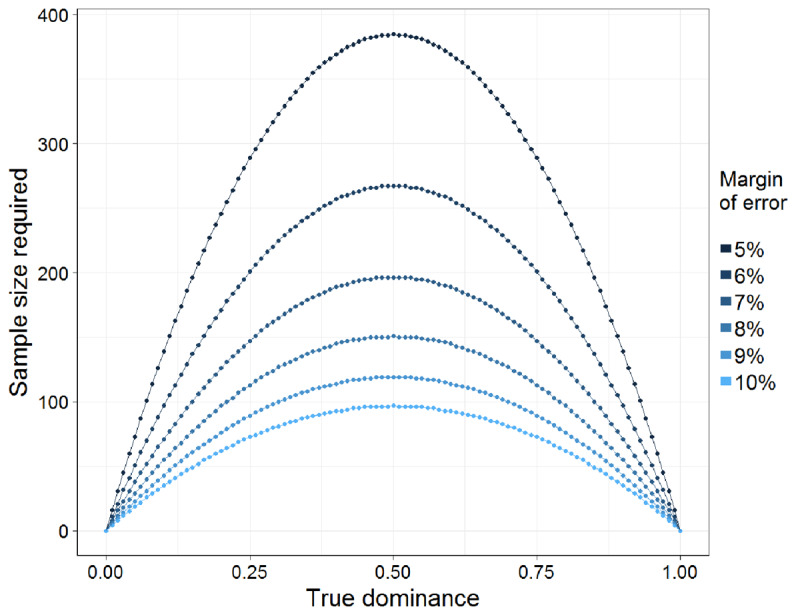
Sample size needed (y-axis) to estimate a dominance value (proportion of responses) with a 95% confidence interval, according to the true meaning dominance value (x-axis) and desired margin of error (colour-coded). More word association responses are needed when the true dominance value is close to 0.5, and for smaller margins of error.

In order to obtain a larger set of target words with at least 100 valid responses per item, we collected more responses for items that had 40–99 responses. The target sample size of at least 100 responses per item was based on a target precision for the dominance estimates. We first computed the required sample sizes needed to estimate the meaning dominance based on a given margin of error, confidence interval, and the true proportion of the most frequent meaning^2^. Across the range of possible true proportions from 0 to 1, the required sample size for estimation (at a given confidence interval and margin of error) forms an inverse U shape, where relatively few responses are needed to estimate very high or very low true proportions, and where more responses are needed to reliably estimate true proportions near 0.5 (see ***Figure 1***). We computed the sample sizes needed across the range of proportions with a 95% confidence interval and a 10% margin of error, and found that these values ranged from 0 responses (for true proportions of 0 and 1) to 97 (for a true proportion of 0.5). Rounding up the required sample size for items with 50% dominance gave us the target of 100 responses per item. We note, however, that the precision (margin of error) of the dominance estimate with a sample size of 100 responses will vary as a function of the item’s true meaning dominance, in that the estimates for biased ambiguous words will be more precise than those for words with balanced meanings. Researchers who require more precise dominance estimates for balanced ambiguous words may wish to collect more word association responses for those items (e.g. 385 responses needed for a 5% margin of error and 50% dominance).

The additional 8880 responses were collected using Qualtrics survey software. Participants met the same inclusion criteria from previous experiments and had not participated in any of the previous word-meaning priming or pre-testing studies. We collected 900 responses (65 items, 30 items per participant) from 30 UCL undergraduates as part of their coursework requirements. The remaining 7980 responses were collected for 114 items from 70 volunteers^3^ who were recruited through Prolific Academic (*https://www.prolific.ac*; Palan & Schitter, 2017). Participants were eligible if they were 18–50 year old native British English speakers, born in the UK and currently residing in the UK. They were paid for their time at the standard hourly rate required by UCL.

Participants first completed the consent process and a language background questionnaire. Then, participants were given the same word association instructions used in previous experiments. They were told that they would hear a series of words, and for each word they should type in an associated word, i.e. a word that is related in meaning. Participants were instructed not to take too long thinking about their response and to just type the first word that came to mind. They were then given some examples of a word and common associates before starting the task. Their responses were coded using the automated coding script. Any responses that were not already present in the existing database were manually coded according to the procedure previously described (see Coding Procedure section).

## Results

The database includes 29,542 valid responses for 243 ambiguous words. The data can be found in the file “RoddGilbert_WA_RawData.csv” (*https://osf.io/r8ucg/*; see “RoddGilbert_WA_RawData_Notes.csv” at *https://osf.io/ngz4p/* for an explanation of the file contents). The mapping between meaning codes and definitions for each item can be found in the file “RoddGilbert_WA_Definitions.csv” (*https://osf.io/4kv8c/*). Note that the meaning code numbers reflect the arbitrary order in which they were added to the database, not necessarily the order of decreasing dominance. Item summary measures, such as response counts and dominance estimates, for all 243 ambiguous words can be found in the file “RoddGilbert_WA_Dominance_Norms.csv” (*https://osf.io/2mduw/*; see “RoddGilbert_WA_Dominance_Norms_Notes.csv” at *https://osf.io/973qr/* for an explanation of the file contents). Finally, the sound files used in all experiments can be found at *https://osf.io/uy47w/*.

**Figure 2:**
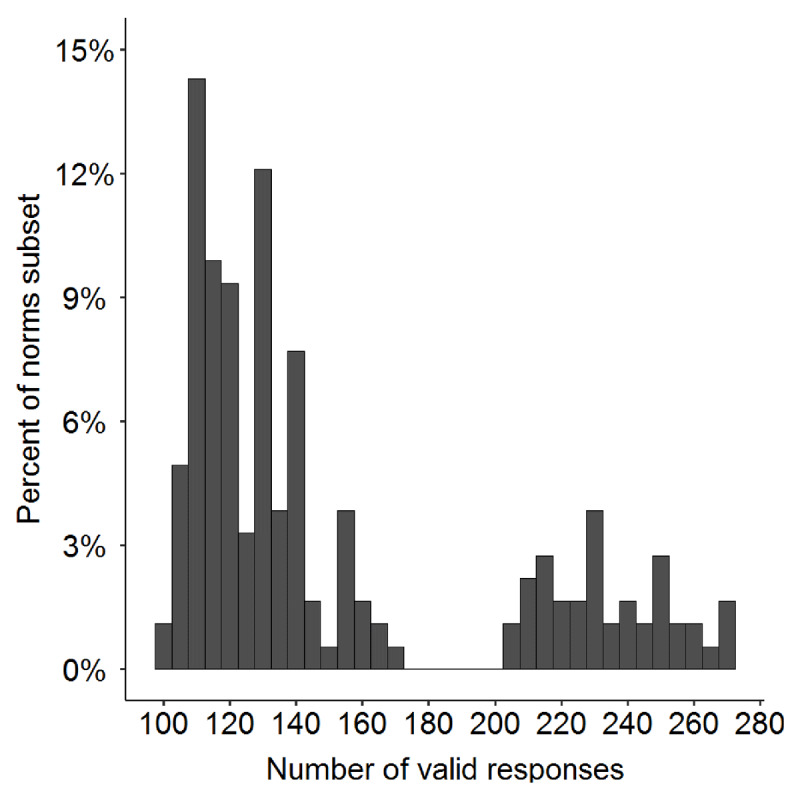
Percentage of items in the norms data subset (y-axis) according to the number of valid word association responses for the item (x-axis). All 182 items in the norms subset have at least 100 valid responses.

From this large database, there were 182 ambiguous words for which we had at least 100 valid responses (M = 151.8 valid responses per item, Mdn = 130, range = 100 – 271; see ***Figure 2***). Of these words, 117 are homographs (e.g., “bonnet”) and 65 are heterographic homophones in British English (e.g., “night”/”knight”, “sauce”/”source”). All the analyses and figures reported here only refer to this smaller subset of 182 items and 27,626 valid responses, which are flagged with “IncludeInNorms = 1” in the item summary file, and we strongly recommend that researchers only use the dominance estimates for these items.

**Figure 3:**
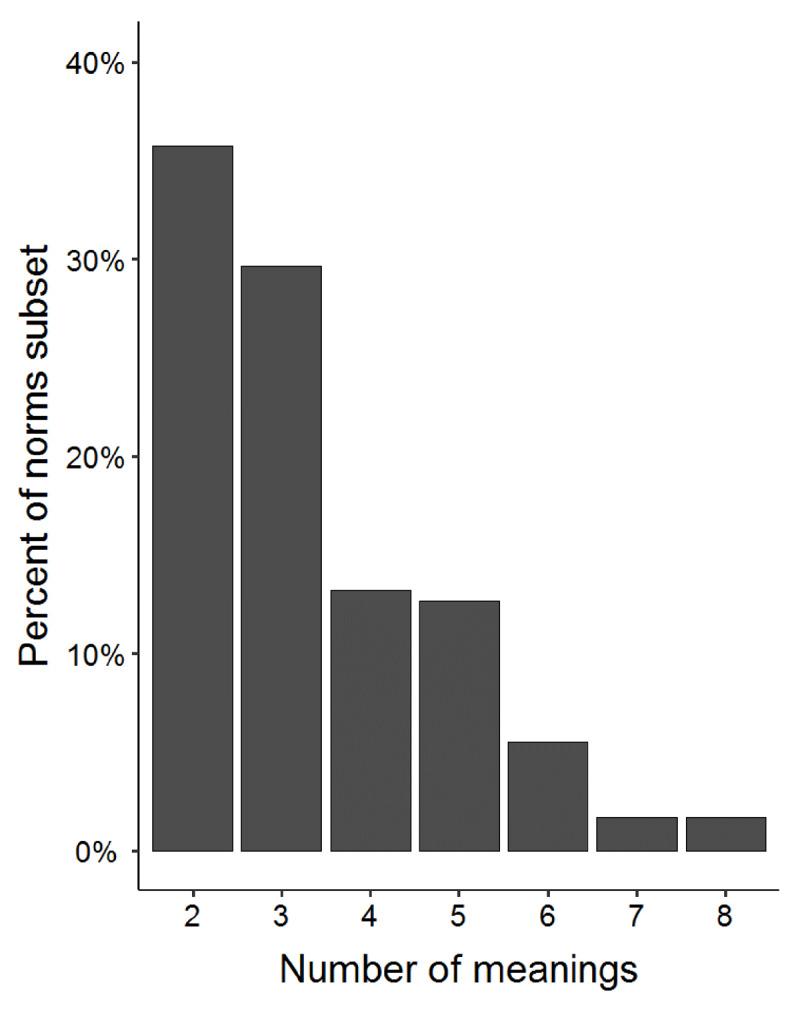
Percentage of items in the norms subset (y-axis) according to the number of meanings for the item (x-axis). All 182 items have at least two meanings.

**Figure 4:**
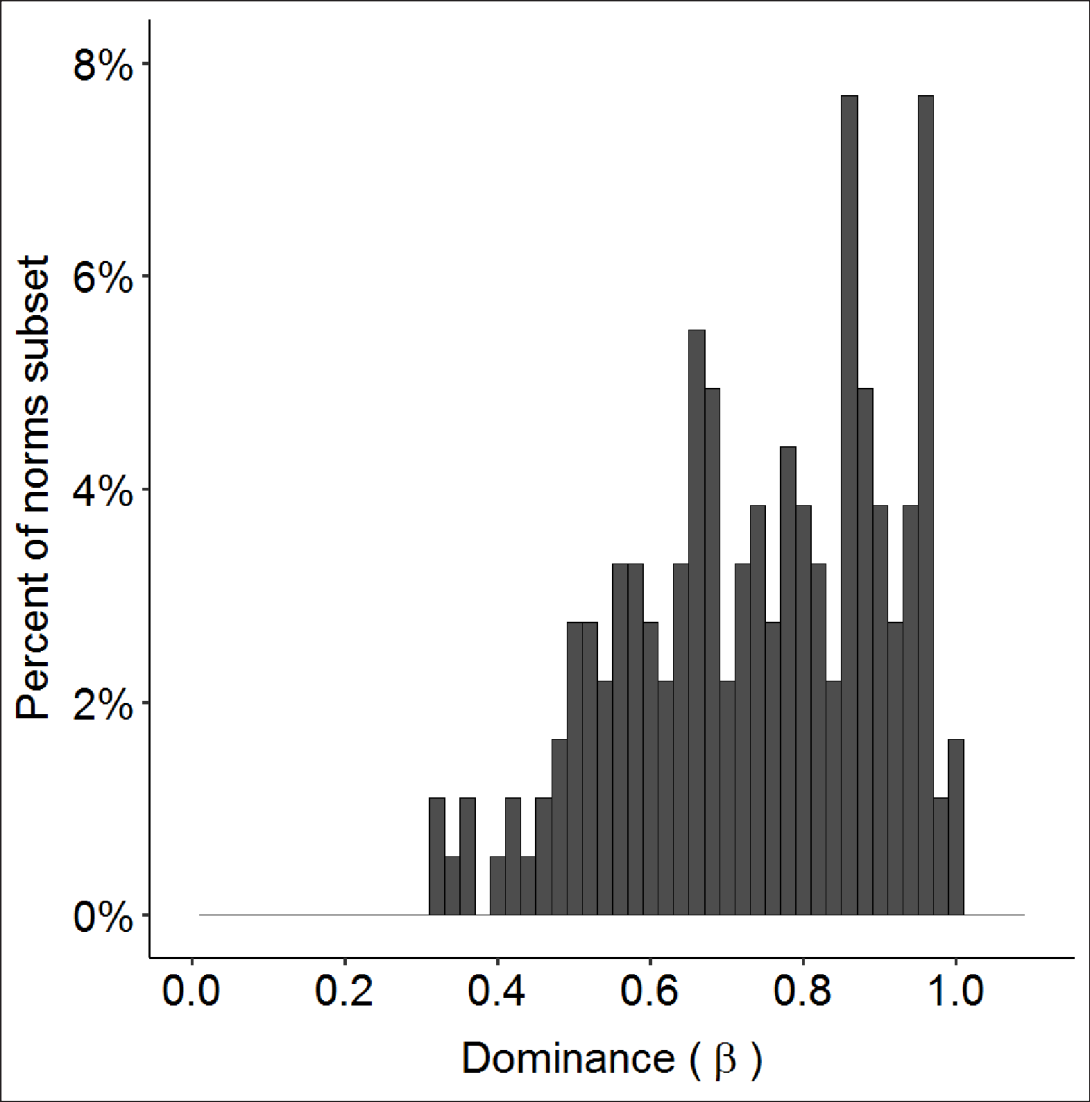
Percentage of the 182 items in the norms subset (y-axis) according to the item’s dominance (proportion of responses for the most frequent meaning; x-axis). Lower dominance values indicate that interpretation is relatively balanced across the different meanings. Dominance values near 1 indicate that interpretation of the word is highly biased toward one meaning.

The number of senses/meanings per item ranges from 2 to 8, with most items having 2 or 3 meanings (see ***Figure 3***). For each item, the relative meaning frequencies were computed by dividing the number of responses corresponding to each meaning code by the total number of valid responses. One simple summary measure of word-meaning dominance is the proportion of responses associated with the most frequent meaning (termed “dominance”, analogous to the β values reported by Armstrong et al. using the eDom method). Values closer to 1 indicate that the item is biased toward one meaning, whereas lower values indicate that interpretation is more evenly split across multiple meanings/senses. Across all items in our set, the mean dominance is 0.74, and ranges from 0.32 (for “pitch”) to 0.99 (for “bed”, see ***Figure 4***).

We calculated two other summary measures for each item: an information-theory uncertainty value (*U*; Twilley et al., 1994) and the standardized difference between the two most frequent meanings (*D*; Armstrong et al., 2012). These measures provide more information about the relative frequencies of an item’s meanings, compared to using the most frequent meaning alone. The *U* value combines the proportions of responses across all meanings to produce a measure of the word’s overall ambiguity using the formula:


U = {\rm{\;}}\mathop \sum \nolimits_{i = 1}^n \;{p_i}lo{g_2}\left( {\frac{1}{{{p_i}}}} \right)

**Figure 5:**
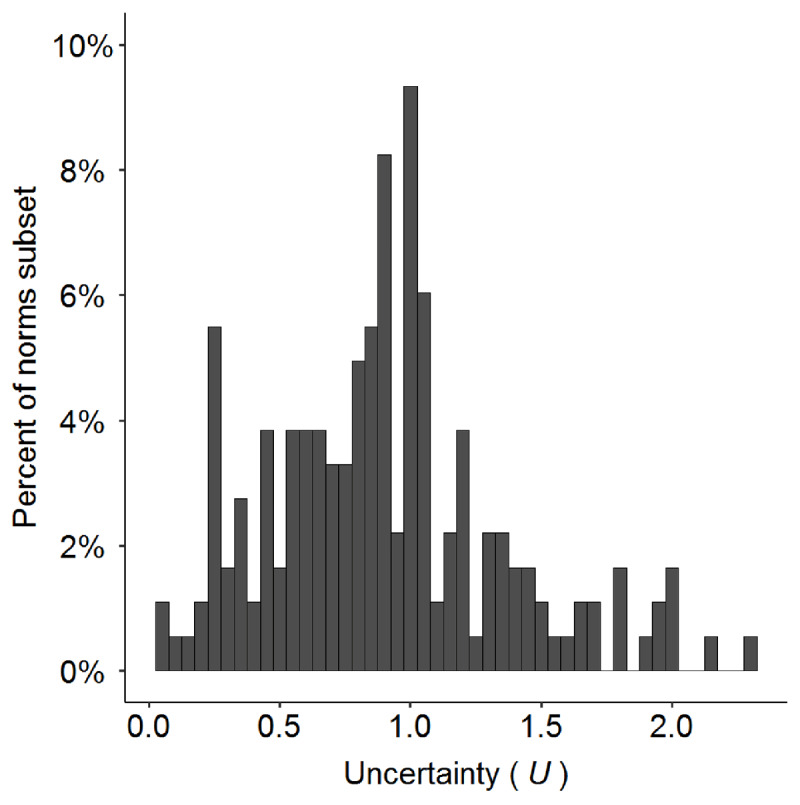
Percentage of the 182 items in the norms subset (y-axis) according to the *U* measure of the item (x-axis), which is a measure of overall ambiguity. *U* values near 0 indicate very low ambiguity, and larger values indicate greater ambiguity.

Where *n* is the number of meanings, and *p*_i_ is the proportion of responses for meaning *i*. The lower bound of *U* values is 0, which indicates very low ambiguity. Higher values indicate greater ambiguity, but with no fixed upper bound, as the *U* value can increase with increasing numbers of meanings. In the present data set, the *U* measure ranges from 0.07 for “bed” to 2.29 for “pitch”. The items are distributed across these values, with more items clustered toward the lower-ambiguity end of the range (M = 0.90, Mdn = 0.89; see ***Figure 5***).

The dominance measure *D* is a standardised difference measure that reflects the degree to which the ambiguous word is biased, that is, when there is one dominant meaning that is much more frequent than the next most frequent meaning, or balanced, when the two most common meanings are relatively similar in frequency. It is computed by subtracting the proportion of responses corresponding to the second most frequent meaning (*P2*) from that for the most frequent meaning (*P1*), and dividing the result by the proportion for the most frequent meaning (*P1*):


D = \left( {P1 - P2} \right){\rm{/}}P1

**Figure 6:**
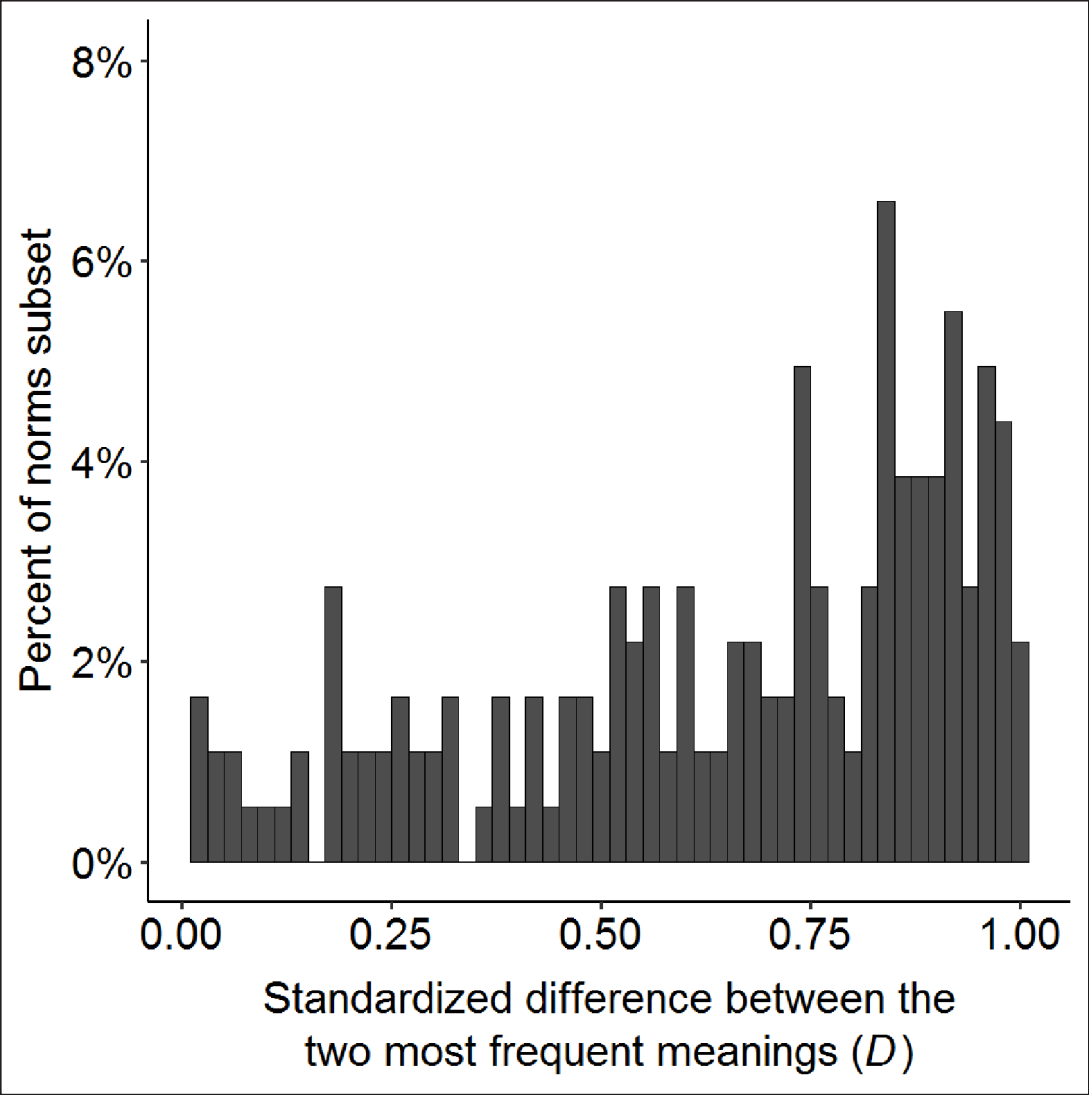
Percentage of the 182 items in the norms subset (y-axis) according to the *D* measure of the item (x-axis), which reflects the degree to which the item is balanced or biased. *D* values near 0 indicate that the item is balanced, i.e. the two most common meanings are similar in frequency. *D* values near 1 indicate that the item is biased, i.e. the most common (dominant) meaning is much more frequent than the next most common meaning.

If a word is perfectly biased (i.e. *P1* = 1.0; *P2* = 0.0) then the *D* measure will be 1, whereas if the word is perfectly balanced (i.e. *P1* = 0.5; *P2* = 0.5) then this measure will be 0. This measure helps to differentiate between items that have similar proportions of responses for the most frequent meaning, but where the remaining proportion of responses is split among several low-frequency meanings (higher *D*, more biased), vs one higher frequency meaning (lower *D*, more balanced). The *D* measure in the present data set ranges from 0.02 (for “board/bored”; *P1* and *P2* both ~0.46) to 0.99 (for “bed”; *P1* = 0.99, *P2* = .01). The words were distributed across the range of *D* values and centered more toward the biased end of the scale (M = 0.65, Mdn = 0.73; see ***Figure 6***).

## Discussion

Most words are ambiguous to some extent, and experiments using these words can provide insight into (i) how people respond to and resolve this ambiguity, and (ii) the structure and plasticity of the lexical-semantic system. However, experiments using ambiguous words must consider the relative frequencies of the different meanings of a word, and use this information as part of the stimuli list construction, experimental manipulations, and/or analyses.

Here we provide a new set of UK-based dominance norms from spoken ambiguous words, based primarily on previously-collected published word association data. We include the raw data, meaning codes/definitions for each item, item summary statistics, and sound files (*https://osf.io/uy47w/*). The item summary data may be useful for researchers who wish to, for instance, select stimuli based on meaning dominance criteria (e.g. biased vs. balanced word sets), match stimuli sets for dominance, or use meaning dominance as a predictor/covariate in their analyses.

The meaning categories for each word were not pre-determined, but rather were developed using an iterative process that required continuous refining in response to participants’ responses. This process provided opportunities to add new interpretations that we had not thought of, including recently-introduced meanings and slang usage, and to split a single category into multiple categories when it was possible to distinguish the interpretations reliably. While our meaning categorisation may not be ideal for all purposes, this fine-grained meaning coding system provides maximum flexibility and allows the researcher to selectively combine and/or exclude meaning categories to suit their needs. An inter-rater reliability analysis using these definitions showed general agreement between our response coding and that of a naïve researcher, along with room for improvement. Our goal is that, by making the definitions and response coding publically available, these will be continuously refined and informed by additional data collection in order to produce clear and empirically-driven categories that can be used to code responses as accurately and reliably as possible.

We also provide an automated coding script for coding new word association responses based on the coded data. This script may help to address one of the main limitations of the word association method, which is the time-consuming nature of the response coding (Armstrong et al., 2012). Our testing shows that around 80% of new word association responses can be automatically coded using this script and so can greatly reduce the time taken to code new responses, while also reducing the number of human errors.

We note that the dominance estimates reported here are not directly comparable to those reported in other norming studies. Each of the aforementioned published norms differs from our dataset in multiple ways, making it difficult to pin-point specific reasons for differences in results. While a comprehensive comparison between this data and previously-published norms is beyond the scope of this report, here we will remind readers of the key differences between this dataset and existing norms, along with some examples.

First, because this data was collected using auditory presentation of the ambiguous words, the heterographic homophone words in our set will be more ambiguous compared to visual presentation, and thus more likely to have different dominant meanings and/or lower dominance estimates. For example, compared to the written word “mail”, the auditory item “mail/male” has an additional lexical interpretation, and consequently has more possible meanings/senses: 3 in our dataset vs 2 in the Armstrong et al. and Maciejewski and Klepousniotou norms. This item also had a different dominant meaning and lower dominance estimate in our norms: .53 for the additional “opposite of female” meaning, versus .83 and .88 for the “letters/parcels sent by post” meaning in the Armstrong et al. and Maciejewski and Klepousniotou norms, respectively. Similarly, in the Twilley et al. data, valid responses to the written word “cell” were fairly evenly split between the “jail” (.55) and “biology” (.45) meanings, whereas the additional “exchange for money” meaning made up the majority of responses (.49) to our spoken “cell/sell” item. Our dataset also includes several heterographic homophones such as “knight/night” that are considered unambiguous in each of the written forms, and therefore not included in norms that use visual presentation.

Second, compared to norms that use data collected from North American participants, the dominance values reported here will reflect differences in dialectical word usage. Some of these differences may be subtle, but there are a few words/meanings that stand out, such as the more commonly British “water spout” meaning of “tap”, which is indeed the dominant meaning in the norms derived from British participants (.87 and .60 in our dataset and Maciejewski and Klepousniotou, respectively), but subordinate for North American participants (.38 in both the Armstrong et al. and Twilley et al. norms). Another example is the “garbage drop-off place” meaning of “tip”, which accounts for around 19% of valid responses in our dataset, but is not listed as a meaning in the Twilley et al. or Armstrong et al. norms (the Maciejewski and Klepousniotou norms did not include the word “tip”). There are also differences in heterographic homophones between these regions due to accent, which changes the possible set of lexical, and therefore semantic, interpretations. For example, the lexical sets “baa/bah/bar”, “court/caught”, “source/sauce” are homophones in southern British English, but are not homophones in many North American accents.

Third, these data have been collected more recently than those reported by Twilley et al., and changes in word usage over time may account for variations from the Twilley et al. norms in particular. Examples from our dataset include the additional internet/social media meaning of “post”, the spreadsheet meaning of “cell”, and the debit/credit payment method meaning of “card”, as well as an increase in the computer-related responses to “bug” (listed as a meaning in Twilley et al. but with .00 proportion of responses, vs .04 in our data).

Fourth, because we have used more fine-grained meaning categories compared with other norms, polysemous words in particular may have a lower dominance estimate in our data set compared to other studies. For example, we have included 7 meanings for “pitch”, with a highest frequency of .32, whereas Armstrong et al. list 2 broader meanings and report a dominance of .88. The dominance estimates for these words could be compared if our meaning categories were collapsed such that they correspond to the same categorisation used in the comparison study – in the case of “pitch”, collapsing our meaning categories to match those from Armstrong et al. would change our dominance estimate to .88 for the same “throw/hurl” meaning. We expected that the dominance estimates for homonymous words with few distinguishable-but-related senses in our set should be comparable with the same estimates from other studies, so long as the same meaning categories were used. In some of these cases, the dominance estimates were indeed similar (e.g. .68, .62, and .58 for the dog-related meaning of “bark” in the Twilley et al., Armstrong et al., and our dataset, respectively), whereas in other cases they differed more than expected (e.g. the cow-related meaning of “calf” was dominant in the Twilley et al. and our dataset, whereas the leg-related meaning was dominant in the Armstrong et al. and Maciejewski and Klepousniotou norms).

The differences between our norms and those that have been previously published raise a number of important questions for future research. For instance, it would be useful to investigate the effects of modality, dialect and other factors on the preferred interpretations of ambiguous words in order to understand whether these factors primarily affect a small set of unique items, such as the examples given here, or if there are perhaps subtle but reliable differences that affect a much broader set of words. Determining the effects of these factors is made more difficult because of the difference in data collection methods (word association and eDom), both of which may systematically under- or over-estimate meaning dominance. In order to answer these questions, the effects of each factor must be examined separately from the norming method, and the norming methods must also be compared against other dominance indicators such as meaning frequencies from corpus studies and predictive validity in task performance (Armstrong et al., 2012, 2016).

We view this database as a dynamic resource. Word association data continues to be collected for ambiguous words for a variety of reasons to address a range of research questions. We encourage all researchers to make use of our coding script to code any new word association data, provide feedback on our meaning categories and response coding, and contact us so that their data can be added to the database in order to expand both the number of ambiguous words and the accuracy and reliability of the dominance estimates.

## Data Accessibility Statement

The raw data, item meaning codes/definitions, item summary statistics, and sound files can be found on OSF at *https://osf.io/uy47w/* (DOI: *10.17605/OSF.IO/UY47W*).

## Footnotes

There are cases where capitalization could potentially aid in disambiguating a participant’s response (e.g. “Polish” vs. “polish”, “China” vs “china). However, we never encountered any cases where capitalization variants would’ve each produced plausible intended meanings for the same target word, and converting to lowercase allows the script to handle participants’ typing idiosyncrasies.

We used the formula: {\rm{n}} \approx \frac{{z_{\alpha {\rm{/}}2}^{2\;}\;\cdot\;p\;\cdot\;\left( {1 - p} \right){\rm{\;}}}}{{{d^2}}} where *z*_α/2_ is the critical value of the Normal distribution at a given alpha level α (e.g. α = 0.05 for a confidence interval of 95%), *p* is the expected proportion, and *d* is the margin of error.

Two additional participants were excluded and replaced during data collection because they responded by typing the spoken word itself, rather than an associate.
